# Induced Autophagy of Macrophages and the Regulation of Inflammatory Effects by Perovskite Nanomaterial LaNiO_3_

**DOI:** 10.3389/fimmu.2021.676773

**Published:** 2021-04-22

**Authors:** Yang Wei, Xuejiao Gao, Feng Zhao, Didar Baimanov, Yalin Cong, Yingying Jiang, Saima Hameed, Yixin Ouyang, Xingfa Gao, Xiaoying Lin, Liming Wang

**Affiliations:** ^1^ College of Chemistry and Chemical Engineering, Jiangxi Normal University, Nanchang, China; ^2^ CAS Key Laboratory for Biological Effects of Nanomaterials and Nanosafety & CAS-HKU Joint Laboratory of Metallomics on Health and Environment, and Beijing Metallomics Facility, Institute of High Energy Physics, Chinese Academy of Sciences, Beijing, China; ^3^ University of Chinese Academy of Sciences, Beijing, China; ^4^ CAS Key Laboratory for Biological Effects of Nanomaterials and Nanosafety, National Center for Nanoscience and Technology, Beijing, China; ^5^ School of Public Health, Qingdao University, Qingdao, China; ^6^ School of Public Health, Capital Medical University, Beijing, China; ^7^ College of Pharmacy, Jilin Medical University, Jilin, China

**Keywords:** perovskite nanomaterials, LaNiO_3_, autophagy, dissolution, inflammation response

## Abstract

Perovskite nanomaterials (NMs) possess excellent physicochemical properties and have promising applications in light-emitting diodes (LEDs), lasers, photodetectors, and artificial synapse electronics. Potential exposure to these NMs happens in the manufacture and application of the perovskite-based products, however, the biological safety of these NMs is still unknown. Here, we used the LaNiO_3_ NM (LNO), a typical kind of perovskite nanostructures to study the interaction with macrophages (J774A.1) and to explore its biological effects at the cellular level. Firstly, we characterized the properties of LNO including the size, shape, and crystal structure using Transmission electronic microscope (TEM), Dynamic lighting scattering (DLS), and X-ray diffraction (XRD). Secondly, to gain a better understanding of the biological effect, we evaluated the effect of LNO on cell viability and found that LNO induced cell autophagy at a concentration of 5 μg/ml and influenced the inflammatory response based on RT-PCR result. Finally, we demonstrated the mechanism that LNO causes cell autophagy and immune response is probably due to the metal ions released from LNO in acidic lysosomes, which triggered ROS and increased lysosomal membrane permeation. This study indicates the safety aspect of perovskite NMs and may guide the rational design of perovskite NMs with more biocompatibility during their manufacture and application.

## Introduction

The crystal structure of perovskite nanomaterials (NMs) can be described as ABX_3_ octahedron (1). The cation of A is composed of lanthanide including La, Sc, Y, Ce, Pr, Nd, Pm, Sm, etc., while the B cation origins from transition element including Ni, Cr, Co, Fe, etc. X is constituted as anion, halogen and chalcogen are the dominating elements (2, 3). Perovskite oxide NMs exhibit more stable and higher electron conduction efficiency compared to conventional NMs with low cost (4, 5), which endows promising application in the fields of energy storage and electronic devices including light-emitting diodes (LEDs), lasers, photodetectors, artificial synapse electronics, wearable electronics, and intelligent vehicles (6–8). During the manufacture and consumption, the latent exposure of such perovskite NMs to human being increases; however, the biological safety of perovskite NMs is still uncertain. Until now, few reports have reported biological effects of the perovskite LNO.

As we know, mononuclear-macrophage system (MPS) is a class of cells including macrophages, dendritic cells (derived from monocytes) and granulocytes (9) that are widely separated in the human body with the property of phagocytosis, in which the cells engulf and destroy bacteria, viruses, and other foreign substances such as nanomaterials (10, 11). As one of the most important barriers, macrophages are responsible for engulfing nanomaterials and removing them *in vivo*. NMs will thus end up in the macrophages of the tissues and organs during their exposure and long-term residence (12, 13). In addition, MPS cells participate in the innate immune responses, through which cells are activated by the NMs and pathogens and then secrete pro-inflammatory cytokines and promote their clearance (14). Thus, it is crucial to evaluate how LNO interact with MPS and the potential effects of LNO on the functions and viability of macrophages after the exposure.

Herein, we investegated the interaction of LNO perovskite oxide NMs with macrophages and potential effects on cytotoxicity and immunological responses. First, we characterized the morphology, size distribution, surface charge, and the component of LNO NMs. Second, we evaluated the effects of LNO on the viability of the macrophage cell line J774A.1 and confirmed that LNO NMs induced cell autophagy. Thirdly, to explore cell autophagy mechanism, we utilized TEM to capture the engulfment, accumulation, and the location of LNO NMs within J774A.1 cells. We also employed inductively coupled plasma mass spectrometry (ICP-MS) to evaluate the release of metallic ions from LNO in an artificial lysosomal fluid (ALF). After the exposure to LNO, we further introduced AO assays by means of Laser scanning confocal microscopic (CLSM) imaging to assess lysosomal membrane integrity (LMP). Finally, we demonstrated that due to the metal ions released from LNO NMs, intracellular Reactive Oxygen Species (ROS) elevated that caused the impairment of LMP. Furthermore, LNO NMs mediated the secretion of cytokines including interleukin, tumor necrosis factor, etc. according to ELISA kit and RT-PCR which suggested immunological effects of LNO NMs. We concluded that LNO-based perovskite materials are chemically active that caused the autophagy of macrophages and mediated immune responses.

## Materials and Methods

### Materials and Chemicals

LaNiO_3_ nanomaterials were prepared according to previous publication (15) and donated by Prof. Hui Wei in Nanjing University. DMEM medium (Hyclone, USA), streptomycin/penicillin(Sigma, USA), Fetal bovine serum (FBS) (BI, Israel), Trypsin-EDTA (TE) (Hyclone, USA), phosphate-buffered saline (PBS) (Hyclone, USA), phenol red-free medium (Hyclone, USA), TBST(Sigma, USA), SDS-PAGE (Hyclone, USA), SDS-polyacrylamide (Hyclone, USA), HNO_3_ (BV-III, Beijing Institute of Chemical Reagents, China), H_2_O_2_ (MOS level, Beijing Institute of Chemical Reagents, China), OsO_4_ (Sigma, USA), and DCFH-DA (Invitrogen, USA). All chemicals were analytical grades and artificial lysosomal fluid was prepared by Milli-Q water.

### Characterization of LaNiO_3_

The morphology of LNO materials were characterized by (TEM, a Tecnai G2 20 S-TWIN) at an accelerating voltage of 200 kV. Zeta potential and hydrodynamic diameter of the materials were measured by a Zeta Sizer Nano series Nano-ZS (Malvern Instruments Ltd., UK); TZY-XRD(D/MAX-TTRIII(CBO) was utilized to acquire the crystal configuration. LNO differences in water and the artificial lysosomal fluids(ALF), which was prepared according to previous publication.

### Cell Culture

Mouse monocytic cell lines or mouse macrophages (J774A.1) were obtained from ATCC, USA. Cells were cultured in a complete medium containing 90% DMEM medium, 10% FBS, and streptomycin (100 μg/ml)/penicillin (100 U/ml) at 37°C in a humidified 5% CO_2_ incubator.

### Cytotoxicity Assay

Cytotoxicity of LNO was determined by a Cell Counting-Kit 8 (CCK-8) assay (Dojindo, Japan). In brief, J774A.1 cells were seeded in 96-well plates at a density of 2.5×10^3^/well overnight. And then, the cell medium was replaced by the fresh DMEM medium containing a series of concentrations (1.25, 2.5, 5, 10, 20, 40, and 80 µg/ml) of LNO. After 24 h treatment, the cells were rinsed twice with PBS at pH 7.4 and further incubated with a 100 µL of mixture including 10% CCK-8 and 90% complete medium (v/v) at 37°C for 1 h in a humidified 5% CO_2_ incubator. The absorbance of the mixture at 450 nm was obtained by a microplate reader (Enspire, USA), while the absorbance of the same sample at 600 nm was used for the reference. Each sample contains five repeated wells for CCK-8 assay.

### Autophagy Fluorescent Probe Analysis

J774A.1 cells were seeded on a petri dish at a density of 5×10^5^ cells/well overnight. After twice rinsing with PBS, the probe for autophagy, DAL Green (Costar, Corning, NY) (16), was incubated with cells for 30 minutes under a 37°C incubator. After twice rinsing with phenol red-free medium, cells were treated with lno(2.5, 5 µg/ml) for 12 h, respectively. Then, a confocal microscope was used to detect the fluorescence signal of DAL Green. The cells were observed under confocal microscope and samples were excited at 488 nm (Perkin Elmer Ultra View Vox system, USA).

### Subcellular Structures Observation by TEM

The cells were seeded in six-well plates and exposed to LNO (5 µg/ml) for 12 h. Then, cells were digested by TE, washed three times with PBS, and collected as cell pellets. About 200 μl of 2.5% (w/w) glutaraldehyde in PBS solution was added to the cell pellets and stored at 4°C overnight. Afterward, the cells were sequentially fixed with 1% (w/w) OsO_4_ in PBS for 1 h, dehydrated with ethanol, embedded in resin, cut into ultrathin sections, placed on the copper grids, and stained with osmic acid before the observation. Finally, the images of the subcellular localization of LNO and the subcellular structures were observed using a bio-transmission electron microscope (HT7700).

### Western Blotting

J774A.1 cells were treated with LNO at 2.5 and 5 µg/ml for 12 h and then lysed in a RIPA lysis buffer (containing protein inhibitor). To obtain the total protein concentration, BCA Kit (Pierce) was employed. For each sample, 20 µg protein was loaded on SDS-PAGE and electrophoresed. The proteins were separated on a 12% or 5% SDS-polyacrylamide gel at 120 V and transferred to a nitrocellulose membrane at 250 mA. At room temperature, the membranes were blocked for 2 h by the solution containing 0.05% Tween-20 (TBST) and 5% non-fat milk that was diluted by TBS buffer. After rinsing with TBST three times, the membranes were incubated with various primary antibodies against β-actin, LC3 I, LC3 II, and p62 (Cell Signaling Technologies, USA) that were diluted by 1:1000, overnight at 4°C. After washing three times with TBST, the secondary antibodies in a blocking solution with a dilution of 1:3000 were added. After 2 h incubation, the membranes were rinsed three times with TBST, followed by chemiluminescence, and finally detected using a gel-imaging analysis system (Bio-Rad, UK).

### Cellular Uptake and Efflux of LNO

To comprehend the process of uptake, the cells were seeded in 6-well plates at a density of 5×10^5^/well and exposed to 5 µg/ml LNO for 3, 6, 12 and 24 h. For the exocytosis, cells were first exposed to 5 µg/ml LNO for 12 h and then rinsed twice with PBS. After that, cells were further cultured for 12, 24 and 36 h in a DMEM medium containing 1% FBS. Each sample had five replicate wells. After rinsing with PBS three time, cells were collected, counted, and centrifuged. Then, the samples were incubated with 4 ml HNO_3_ overnight within conical flasks. Next, the samples were heated for 2 h with a temperature maintaining at 150°C During the heating, the 30% H_2_O_2_ was gradually added to the flasks drop by drop until the solution became colorless. Afterwards, the solution was cooled to room temperature and diluted by a 2% HNO_3_ solution to a final volume of 3 ml. A series of concentrations containing 0.1, 0.5, 1, 5, 10, 50, 100, 500, and 1000 ng/ml lanthanum and nickel were prepared as standard solutions. In addition, a final concentration of 40 ng/ml indium was added as the internal standard. All the solutions were measured three times by ICP-MS (Thermo, USA) and the data were shown as mean value and standard error (17–19).

### Detection of Metal Ion Release in ALF

LNO at 200 µg/ml (10 ml) were incubated with the ALF with a pH value of 4.5 for 3, 6, 12 and 24 h, respectively. After different incubation time, the sample were collected by a centrifugation at 9000 rpm for 15 min. The supernatant was digested according to the procedure mentioned above and diluted by a 2% HNO_3_ solution to a final volume of 3 ml before the measurement by ICP-MS. The ratio of La and Ni was used to evaluate the ion release.

### Analysis of Lysosomal Membrane Integrity

Acridine orange (AO, Sigma, USA) assay (20) was used to assess the lysosomal membrane integrity (21). Cells were first seeded in a 6-well plate at a density of 5×10^5^ cells/well, stained with 1 µg/ml AO for 15 min, rinsed with PBS, and then exposed to 5 µg/ml LNO for 12 h in a complete medium. Then rinsed with PBS, dispersed in a serum-free and phenol red-free medium. Finally, the cells were observed under confocal microscope (Perkin Elmer Ultra View Vox system, USA) and samples were excited at 488 nm, and emission was detected at 537 nm (green) and 615 nm (red).

### Reactive Oxygen Species (ROS) Assay

Cells were seeded on a petri dish with a density of 5×10^5^ cells/well. After 24 h culture, cells were exposed to 2.5 and 5 µg/ml LNO for 12 h. After twice rinsing with PBS, the cells were incubated with PBS containing 10 µM DCFH-DA for 25 min at 37°C. After twice rinsing with PBS, cells were cultured with serum-free and phenol red-free medium and then observed by a microscope with the excitation wavelength at 488 and the emission at 525 nm.

### Real-Time Reverse Transcription Quantitative PCR (Real-Time RT-qPCR) and Inflammation Effects

Cells were differentiated onto 6-well plates at a density of 1×10^5^ cell/well for 48 h. To prime the macrophages, the cells were treated with 5 µg/ml lno for 12 h. RT-PCR experiments were done to determine the level of mRNA expression. The TRIZOL reagent method (Life Technology, CA, USA) was utilized to isolate RNA from cells. About 10 p mol oligonucleotide (Oligo dT)(Sigma, USA) primer and Moloney murine leukemia virus reverse transcriptase (M-MLV, Promega, Madison, USA) added 2 μg of RNA was used to generate cDNA. Each sample was prepared for real-time quantitative PCR in a final reaction volume of 20 μl by adding Master Mix (Promega, Madison, USA) and SYBR Green (Invitrogen, Paisley, UK). The amplification cycle was performed by Realplex4 (Eppendof, Germany). The primers synthesized by Sangon Biotech (China) were shown as below:

TNFα:

F-CATCTTCTCAAAATTCGAGTGACAA
R-TGGGAGTAGACAAGGTACAACCC

IL10:

F-CTTACTGACTGGCATGAGGATCA
R-GCAG CTCTAGGAGCATGTGG

IL-1β:

F-TGAAATGCCACCTTTTGACAGTG
R-ATGTGCTGCTGCGAGATTTG

NF-κB:

F-GGGCTATAATCCTGGACTTCTGG
R-AGTTTCCAGGTCTGATTTCCTCC

IL-8:

F-CACCTCAAGAACATCCAGAGT
R-CAAGCAGAACTGAACTACCATCG

IL-6:

F-GAGGATACCACTCCCAACAGACC
R-CAAGCAGAACTGAACTACCATCG

GAPDH:

F-GACCCCTTCATTGACCTCAAC
R-CTTCTCCATGGTGGTGAAGA

### Statistical Analysis

All the data were statistically analyzed using Origin 9 software by one-way ANOVA or Student *t* test. Significant difference based on that *p* value is less than 0.05. Data were shown as mean value ± standard error of three replicated experiments at least.

## Results

### Characterization of LaNiO_3_

**Figure 1:**
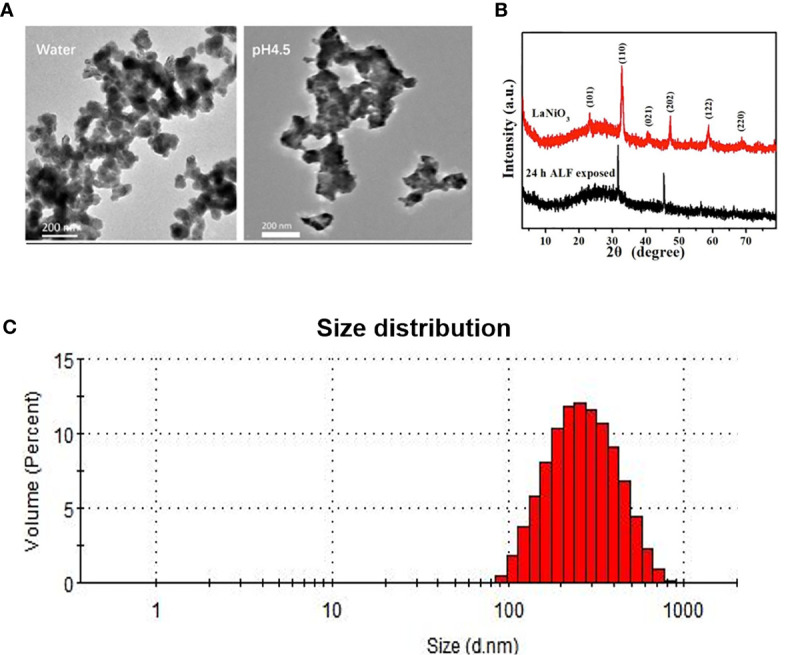
Characterization of LNO. **(A)** TEM images of LNO NMs as dispersed in water and ALF. **(B)** Powder X-ray diffraction patterns of LNO NMs as dispersed in water (red line) and ALF (black line). **(C)** Size distribution of LNO NMs in water as determined by DLS.

TEM was employed to characterize the morphology of LNO in water and artificial lysosomal fluids (ALF) at pH 4.5 as shown in **Figure 1A**. The result indicated that there was structural variation in LNO NMs from the water to the ALF after 24 h exposure LNO displayed long tentacle-like shape in ALF rather than in the water, which meant physical or chemical changes in LNO within the acidic ALF environment. This result was further confirmed by XRD results. Compared with XRD pattern of LNO NMs dispersed in water, the characteristic peaks for 101, 021, 122 and 220 crystal facets disappeared in the ALF (**Figure 1B**), which suggested the impaired structure of LNO in the acidic lysosome. Based on DLS measurement, we found that the average of hydrodynamic size of LNO NMs in water was 350 ± 20 nm (**Figure 1C**).

### Cytotoxicity and Cellular Location of LaNiO_3_

**Figure 2:**
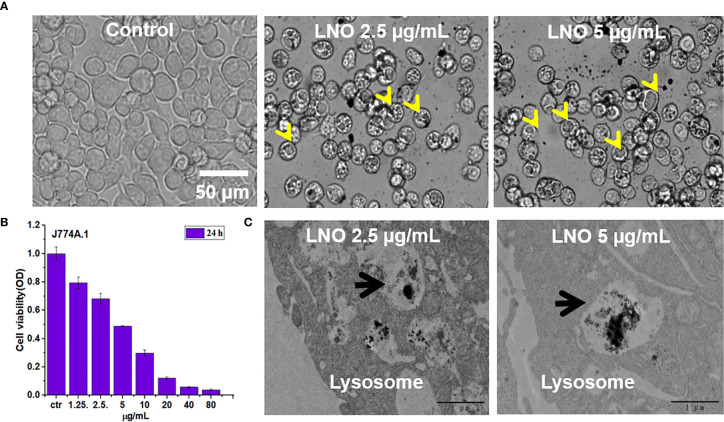
Cytotoxicity and localization of lno nms. **(A)** Images of j774a.1 cells before and after treatment with 2.5 and 5 µg/ml LNO as observed by an optical microscope. **(B)** Dosage-dependent effects of LNO on J774A.1 viability after 24 h exposure as determined by cck-8 assay (*n*=6). **(C)** TEM images of subcellular structures and intracellular location of LNO after 24 h treatment. The arrows indicate the lysosomes.

**Figure 3:**
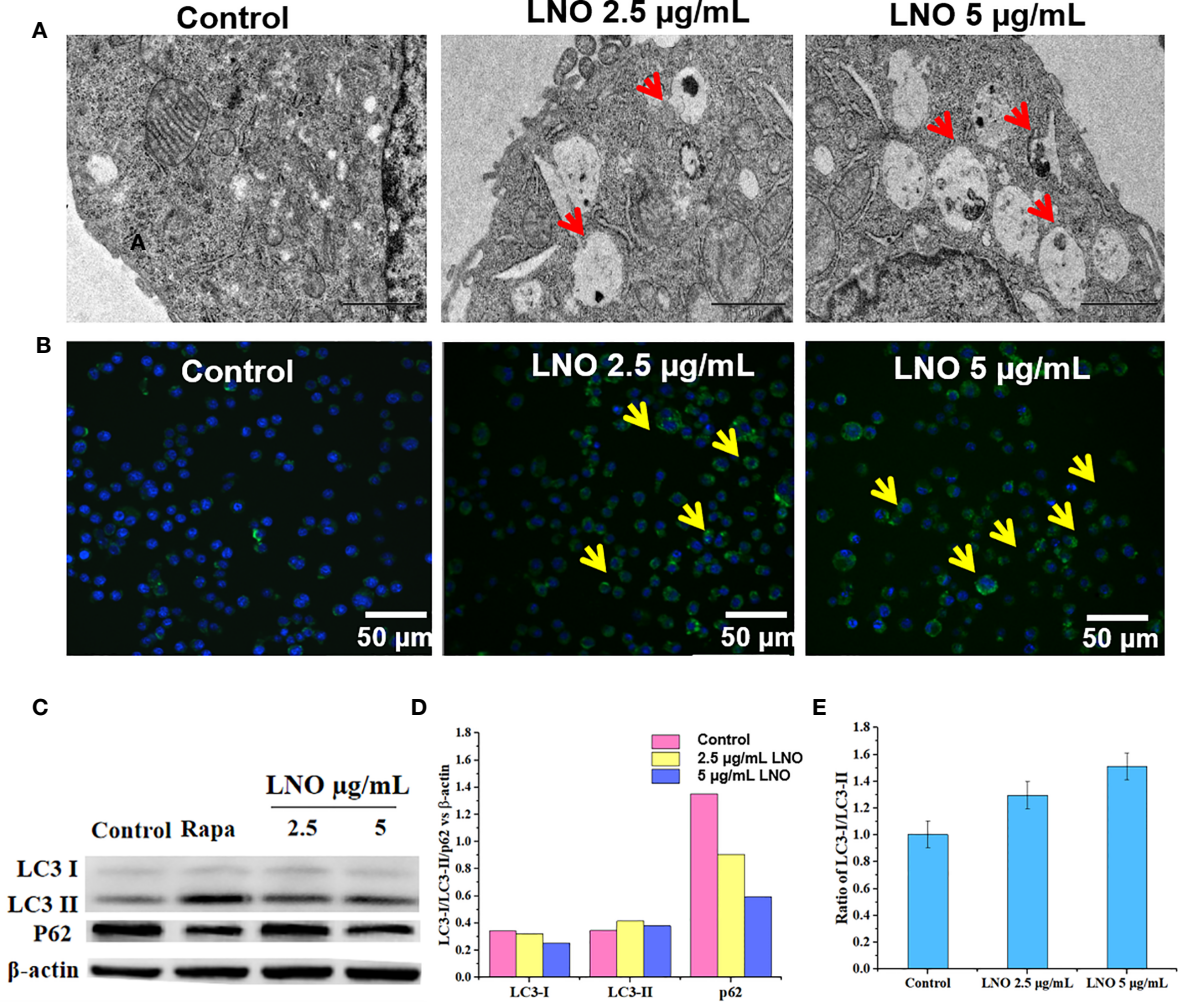
Cell autophagy induced by LNO. **(A)** TEM images for the subcellular structures indicating the induced autophagolysosome in J774A.1 cells after 12 h exposure to 5 µg/ml LNO. **(B)** Images of the autophagy-specific fluorescent probe when J774A.1 cells were exposed to LNO for 12 h and labeled with the probes as observed by CLSM. **(C)** The expression of autophagy-related proteins including lC3-i, lC3-ii, and p62 in J774A.1 cells after LNO exposure for 12 has detected by western blotting. **(D)** The ratio of intensity of the lC3-i, lC3-ii, and p62 protein expression compared to β-actin as calculated by the gray value according to Image J software. **(E)** The ratio of the lC3-ii VS lC3-i *expression* calculated by the gray value according to Image J software.

We then investigated the effect of LNO on the viability of j774a.1 cells after 24_ h_ exposure (**Figures 2A, B** **)**. CCK-8 results indicated that LNO NMs decreased cell viability in a dose-dependent manner with an IC50 concentration of 5.08 ± 0.14 μg/ml (**Figure 2B** **)**. For the following experiments, the concentrations of 2.5 and 5 µg/ml were chosen to evaluate the cellular effects of LNO NMs. Compared with the control, multiple and large intracellular vesicles with a size of several micrometers (labeled by yellow arrows) formed after LNO NMs treatment, which were observed by optical microscope (**Figure 2A**). TEM images show the formation of large vesicles and the accumulation of LNO NMs in the lysosomes or the phagolysosomes. Within the organelles, the most of LNO aggregated. At a higher dosage of 5 µg/ml, the exposure of LNO NMs resulted in much more and larger intracellular vesicles compared to the control and that at 2.5 µg/ml. In addition, the mitochondrial structure turned to be swelling (**Figures 2C**, **3A**). Thus, TEM results suggest that LNO NMs might influence the structure of organelles such as lysosomes, phagolysosomes, and mitochondria.

### Autophagy Induced by LaNiO_3_

We further checked death pathways of LNO-treated for J774A.1 cells at the morphological and molecular levels. Autophagy plays a crucial role in the processes of removal, degradation, and recycling of misfolding proteins or damaged organelles. When cells sense the signals for misfolding proteins and damaged organelles, the autophagy is triggered and the autophagosomes will form that is a type of vesicles composed of double-layer membrane with engulfed matters (22). In an acidic environment, autophagosomes fuse with lysosomes to form autophagolysosomes, and the contents of autophagolysosomes will be degraded by digestive enzymes in the lysosomes (23). After the exposure to LNO at the sub-lethal doses such as 2.5 and 5 µg ml^−1^ for 12 h, both the autophagosomes and autolysosomes were visible based on TEM images (**Figure 3A**).

In addition, DAL Green is a small-molecule probe that is specific for autophagy, which is capable of emitting fluorescence in hydrophobic and acidic environments and can be thus used to identify autophagolysosomes (24). After the exposure to 2.5 and 5 µg ml^−1^ LNO for 12 h, obvious fluorescence signal appeared compared to the control that suggested the induced autophagic lysosomes by LNO (**Figure 3B**).

Moreover, we verified the autophagy at the protein level by analyzing the conversion of the autophagy-related protein, microtubule-associated protein 1 light chain 3 (LC3). LC3 has two isoforms: LC3-I is cytosolic, while LC3-II is associated with autophagosome membranes. Autophagy is featured as an increase ratio of LC3-II protein (25). WB results show that both the level of LC3-II and the ratio of LC3-II to LC3-I expression enhanced with the increasing dosage of LNO (**Figures 3C, E** **)**. As we know, p62, or SQSTM1/sequestosome1, is a substrate that is preferentially degraded during the autophagy (26). The expression of p62 was downregulated with the increasing LNO concentration (**Figures 3C, D** **)**. Taken together, LNO induced the autophagy of J774A.1 cells.

### Impairment in Lysosomal Membrane Integrity Induced by LNO

**Figure 4:**
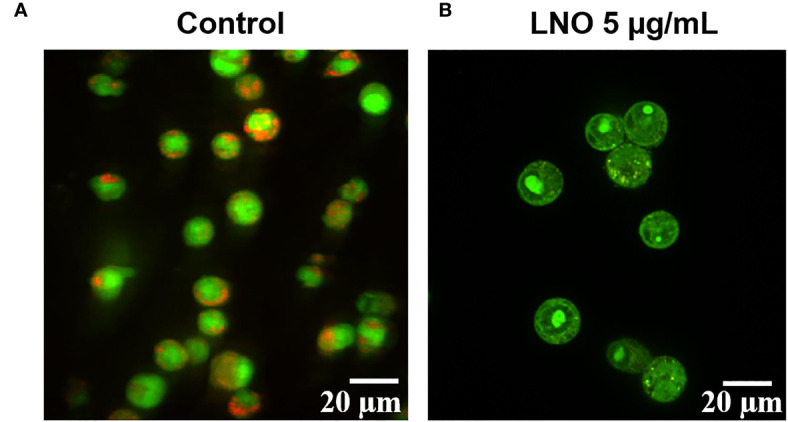
Influence of LNO on the lysosomal membrane permeation. The lysosomal membrane integrity of J774A.1 before **(A)** and after the exposure **(B)** to 5 μg/ml LNO 12 h using AO staining. The scale bar represents 20 μm.

Acridine orange (AO) is a pH-sensitive probe for that is proper for the assessment of lysosomal membrane integrity. AO can penetrate cell membrane and distributes in the different organelles where it exhibits distinct fluorescence signals. With an excitation at 488 nm, at the acidic environment such as lysosomes/endosomes, AO emits bright red color while it shows green color at the neutral or basic environment such as the cytoplasm and the nucleus. When the lysosomal membrane integrity decreased, the pH will increase and then there was significant color change in the lysosome from red to green or yellow (27). Confocal images for AO staining show that LNO increases lysosomal membrane permeation at 5 µg/ml, in which the fluorescence of lysosomes changed from red to green (**Figure 4**).

### Release of La and Ni Ions in the Acidic ALF Environment

**Figure 5:**
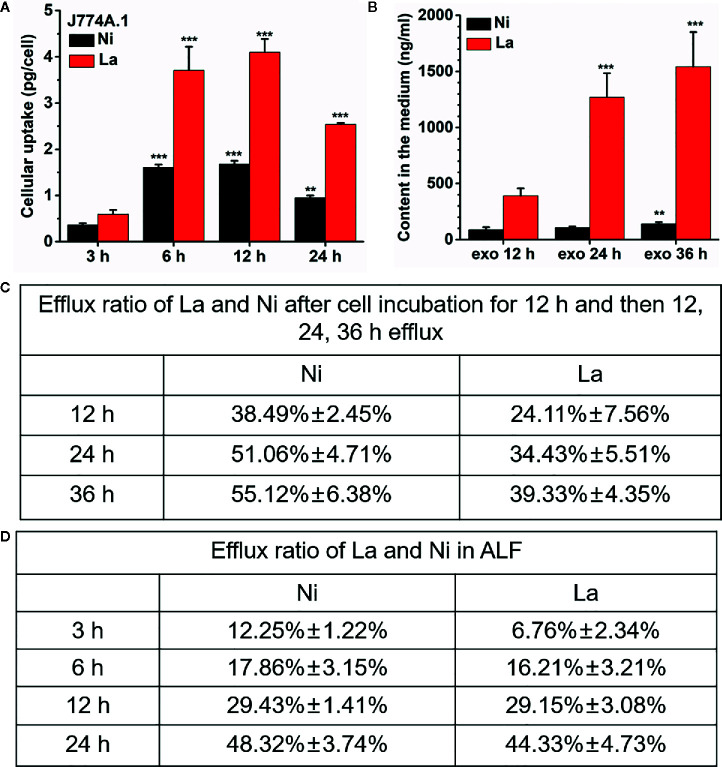
Cellular uptake of LNO and the release of La and Ni from LNO. **(A)** The uptake of LNO in J774A.1 cells after exposure to 5 µg/ml LNO as determined by ICP-MS (*n*=5). The signs (** and ***) indicates significant different for the level of La and Ni between the group and that at 3h uptake with p < 0.01 an p < 0.001, respectively. **(B)** The exocytosis of La and Ni elements in the supernatant as detected by ICP-MS. Cells were exposed to 5 µg/ml LNO for 12 h and then further cultured in fresh medium for 36 h (*n*=5). The signs (** and ***) indicates significant different for the level of La and Ni between the group and that at exo 12h with p < 0.01 an p < 0.001, respectively. **(C)** The percentage of the released La ions and Ni ions in J774A.1 cells that was calculated by the formula of the amount of element released/the uptaken element amount. **(D)** The persistent release of metal ions from LNO when LNO was incubated with ALF solution as detect by ICP-MS (*n*=5). The sign *** indicates the very significant difference between samples and control (p < 0.001).

The degradation and dissolution of perovskite materials in the organisms and the release of metal ions is a crucial way to induce toxicity (28, 29). To understand why LNO influence the lysosome membrane, it is necessary to explore chemical behaviors of LNO in the lysosomes. We then used ICP-MS to determine the release of La and Ni ions within the artificial lysosomal fluid. After the exposure to LNO for 24 h at 5 µg/ml, the amount of intracellular lanthanum and nickel ions increased in a time-dependent manner within 12 h while it decreased at 24 h, suggesting the uptake reached an equilibrium at 12 h and cells removed LNO or metal ions after the uptake (**Figure 5A**). The accumulation of La was much higher than that of Ni in the cells, which may be due to much more removal of Ni than La during the internalization of LNO. After 12 h uptake, time-dependent removal by cells was evaluated after the withdraw of LNO in the cell culture media. ICP-MS results indicated that both La and Ni were released in the media with the increasing time (**Figure 5B**). We calculated the percentage of the metal ions released from the accumulated LNO at 12 h uptake and found that the efflux ratio of Ni was much higher than La during 36 h exocytosis, which suggested faster removal of Ni than La during the cell-LNO interaction (**Figure 5C**). The reason may be that a part of La ions may form LaPO_4_ in the cytoplasm that decreased the exclusion process, while Ni can be excluded by cells by means of metal ion transporters and pump (30, 31).

Furthermore, we evaluated the dissolution of LNO and the release of ions in ALF fluid with a pH of 4.5. When LNO NMs were centrifuged and the supernatant was collected to quantify the dissolved metal ions from LNO. After continuous incubation of LNO with ALF for 36 h, the released La and Ni from 200 µg/ml LNO suspension was shown in **Figure 5D**. At such an acidic buffer, LNO gradually released metal ions with the time and the dissolution rate for La and Ni kept the same.

### Triggering High Level of Oxidative Stress by LNO

**Figure 6:**
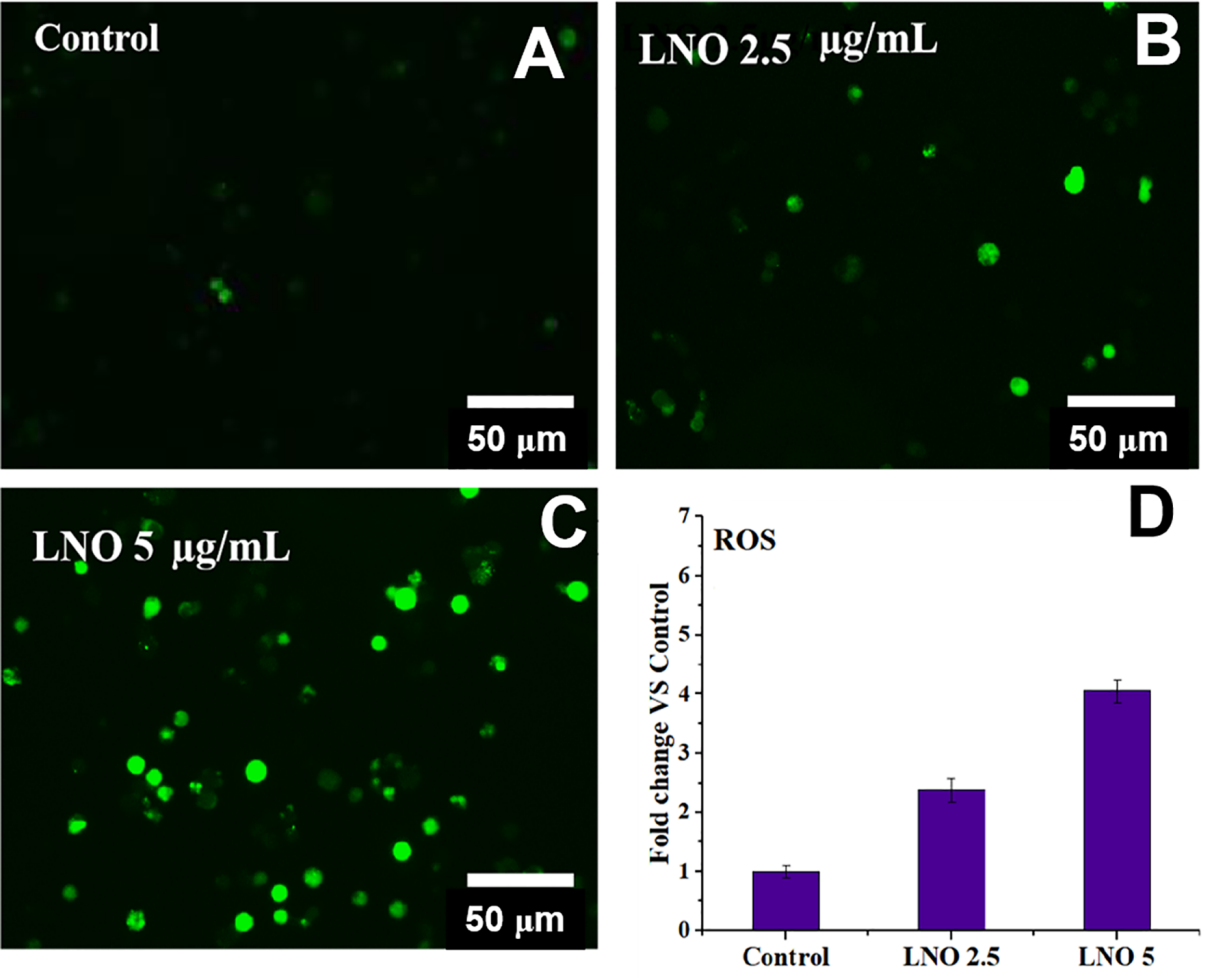
Intracellular ROS level after the exposure to LNO. **(A–C)** Optical images for intracellular ROS stained by DCFHDA when cells are treated by 2.5 and 5 µg/ml LNO for 12 h. **(D)** Quantitative analysis of relative intensity of intracellular DCF fluorescence based on optical images. Data are expressed by mean value and standard errors (*n*=3).

To verify the effects of metal ions derived of LNO, we evaluated the production of ROS in J774A.1 cells after the exposure to LNO. After the incubation with 2.5 and 5 µg/ml LNO, DCF, a ROS-specific fluorescent probe was used to determine the level of ROS in J774A.1 cells at 12 h. Optical microscopic images show that LNO significantly promotes the production of ROS in a dose-dependent manner (**Figure 6**). After the exposure to 2.5 and 5 µg/ml LNO, intracellular ROS levels separately elevated to ~2.2 and ~3.2 folds compared to control, which may be explained by the uptake of LNO and the release of metal ions. As we know, transition metal ion may cause oxidative stress by interacting with antioxidant systems in the cells and taking part in catalytic reactions (32). Oxidative stress refers to the imbalance between oxidation and antioxidation in the body, which can be caused by the massive production of reactive oxygen species (ROS) that tends to oxidize biological molecules, leads to inflammatory infiltration of neutrophils and increases secretion of proteases. ROS directly participates in the regulation of cell survival and death (33–35). The overloaded free radicals can oxidize proteins, lipids, and nucleic acids within cells and impair their structures and functions. In the case of high level of oxidative stress, the damage in the organelle membrane structure such as proteins/enzymes and phospholipids may increase LMP and trigger the release of cathepsin B and D from lysosomes. As a result, the damage in lysosomes/endosome membrane promotes the release of lysosomal contents that activates cell autophagy (24).

### Promoting the Immune Responses by LNO

Cytokines include blood cell growth factors and interleukins, abbreviated as IL that refers to the lymphokine, which are involved in the cell-cell interaction for white blood cells and immune cells in order to regulate immune responses (36, 37). Cytokines play crucial roles in the activation and regulation of immunological system, which mediate the activation, proliferation and differentiation of T and B cells, and also affect inflammatory responses (38, 39). The activation of nuclear factor kappa beta (NF-κB) pathway is an upstream event that mediates inflammatory responses, which may be involved in the occurrence and development of multiple diseases (40). Both the increased lysosomal membrane permeation and the autophagy are related to immunological effects of macrophages (41, 42). We thus tested the expression of inflammatory response-related factors including tumor necrosis factor-α (TNF-α), Interleukin-6(IL-6)/Interleukin-1β (IL-1β), and NF-κB by RT-PCR when the cells were treated with 2.5 and 5 µg/ml LNO for 12 h.

**Figure 7:**
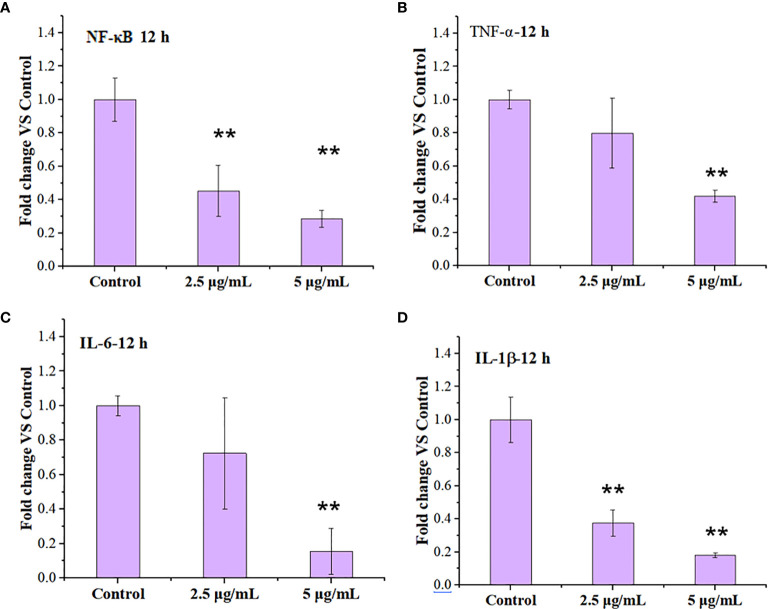
Regulation of immune response in the macrophages by LNO. The expression of NF-κB **(A)**,TNF-α **(B)**, IL-6 **(C)**, and IL-1β **(D)** in J774A.1 cells after the exposure to 2.5 and 5 µg/ml LNO for 12 h as measured by RT-PCR (*n*=3). The sign ** indicates the significant difference between the LNO-treated group and the control.

RT-PCR results show that LNO at 5 µg/ml suppressed the expression of NF-κB after 12 h treatment (**Figure 7A**). Moreover, the expression of proinflammatory cytokines including TNF-α (**Figure 7B**), IL-6 (**Figure 7C**), and IL-1β (**Figure 7D**) at mRNA level was decreased after the LNO treatment. The gene expression of these factors at mRNA level thus indicated that LNO exhibited immunosuppressive effect by inhibiting NF-κB pathway and the expression of several inflammatory factors (**Figure 7**). For classical NF-κB pathway, NF-κB molecule enters the nucleus through p65 and p50 to regulate inflammatory activation which can be induced by cytokines, ROS, and metal ions etc. However, selective autophagy can modulate p100/p52 stability that inhibits the activation of non-canonical NF-κB pathway (43), by which we can understand the reason why LNO induced autophagy efficiently suppressed the inflammatory responses. Moreover, TNF-α is mainly secreted by macrophages and plays an important role in the onset of inflammation (44), which and involved in the regulation of tumor microenvironment and the development in diseases (45). IL-6 can be produced by macrophages and has pleiotropic functions in immune system, which activates immune cells to remove pathogens, to repair damaged tissues, to regulate acute immune response (46) and is also involved in autoimmune diseases and chronic inflammation (47). As an inflammatory cytokine, IL-1β is widely involved in a variety of pathological damage processes such as human tissue destruction and edema formation as well as inflammation response (48, 49). In summary, the decreased expression of proinflammatory cytokines suggests that LNO may serve as a potential inhibitor for the inflammation therapy.

## Discussions

The study about the dissolution and release of heavy metal ions from LaNiO_3_ is crucial to understand the safety of LNO perovskite nanomaterial. The toxic effects of metal ions on tissue and cells are well-known in many studies (28, 29). The quantitative results for intracellular La and Ni by ICP-MS show that both of La and Ni can be internalized by macrophages quickly and then a half of them can be released at least within 24 h. These metal ions contribute to the lysosomal membrane impairment. One of the major reasons is that the released La ions from LNO can react with the phosphate groups of phospholipid heads on the lysosomal membrane (50), which probably destroys the structure of the lysosomal membrane and triggers autophagy. In addition, the released Ni ions may also contribute to ROS production through Fenton-like catalytic reaction.

Under the exposure to stressors such as pathogens and inflammatory signals, autophagy plays crucial roles in innate and adaptive immunity as an effector and mediator, during which autophagy-related proteins act to achieve a balance between activation and inactivation of innate immune signaling (51, 52). For example, the formation of NLRP3 inflammasomes can be suppressed due to the degradation of NOD-like receptor (NLR), a key component of NLRP3 inflammasomes during the autophagy As a result, the maturation and secretion of IL-1β and IL-18 decreased and the inflammation is suppressed (52, 53). In addition, under the stimulation by the stressors, pathogen-recognition receptors (PRR) such as toll-like receptor (TLRs) can form a complex with MyD88 and TRIF but are recognized by autophagy-related receptors, such as SQSTM1, HDAC-6, NDP52, which triggers the degradation of the TLR-containing complex in the autolysosomes (54). The other autophagy-related receptors, such as OPTN and UBQLN1, can promote the degradation of TRIF and TRAF in the autolysosomes. As a result, TLRs signaling is negatively regulated in the autophagy (52, 53). Thus, LNO can not only induce macrophage autophagy but also is capable of suppressing inflammation.

Macrophage autophagy regulates the physiological and pathological process. For example, autophagy can targeted degrade IL-1β, inhibits the activation of inflammasome NLRP3 and reduces the release of inflammatory cytokines. Induced autophagy may weaken sepsis inflammation according to immunosuppression in the late stage of sepsis to a certain extent (36). In addition, macrophage autophagy plays a vital role in the physiological functions of the pulmonary system and its inflammatory response during the infection, pathogenesis, and chronic lung diseases. In a mouse model of acute lung injury induced by hemorrhagic shock, the autophagy of macrophages can inhibit inflammation and reduce acute lung injury (37). By starvation or rapamycin treatment, the autophagy of macrophages promotes the elimination of Mycobacterium tuberculosis (55). Furthermore, macrophage autophagy has been shown to be highly related to the regulation of intestinal natural immune response. AMPK activator GL-V9 can trigger macrophage autophagy, degrade NLRP3 inflammasomes, and have a protective effect on colitis and tumor formation in mouse colitis-related colorectal cancer (56, 57). Therefore, based on the capability of inducing autophagy of macrophages, LNO may serve as a nanomedicine to inhibit inflammation for the therapy purpose in immune regulation diseases, such as tumors, lung injury, and intestinal immune diseases.

## Conclusion

**Figure 8:**
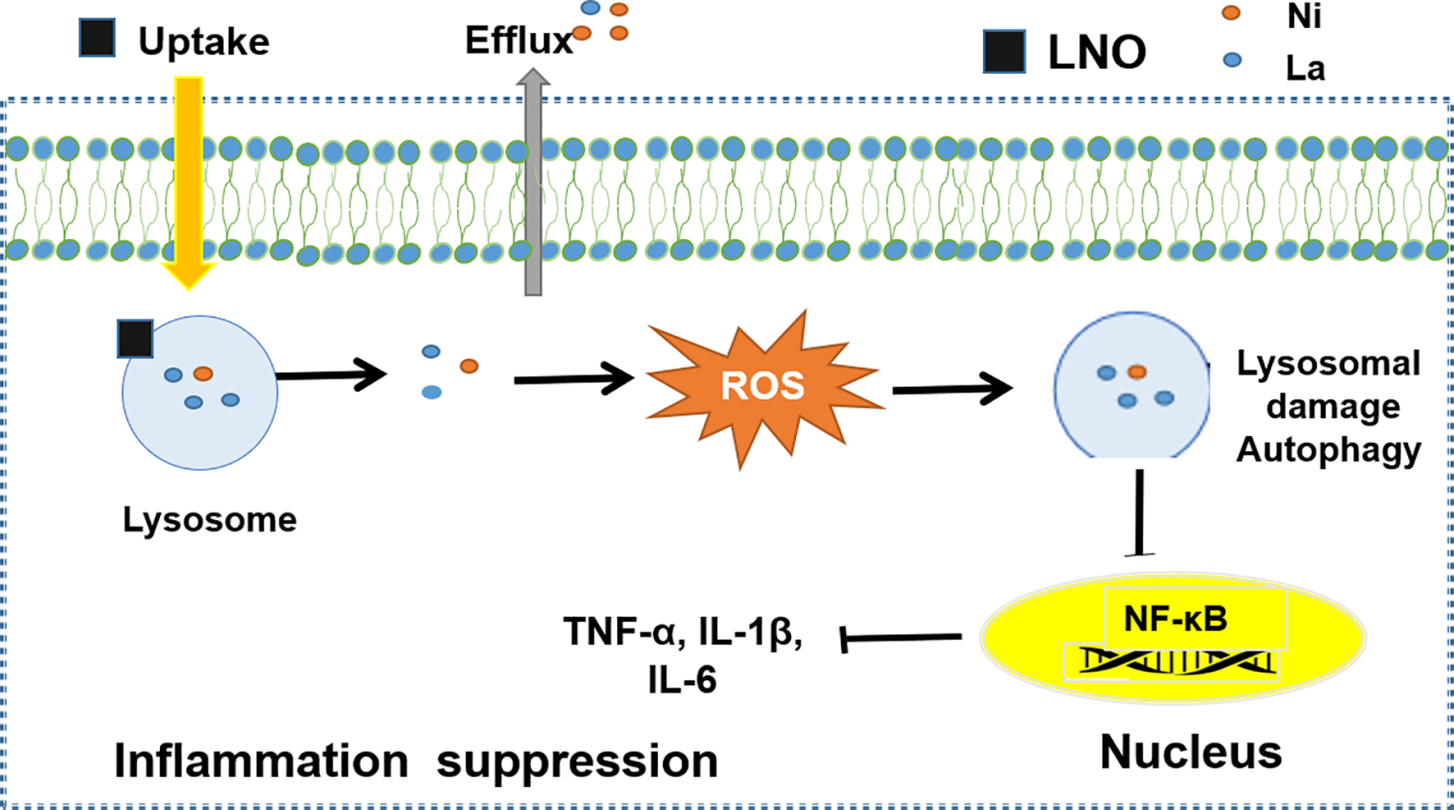
The mechanism of LNO induces protective autophagy of monocytes by inhibiting the expression of inflammatory factors to promote immune response.

In summary, we studied the interaction of a typical perovskite LaNiO_3_ (LNO) with the macrophages and biological effects. We found that LNO nanomaterials reduced the viability of macrophages *via* the autophagy and show the autophagy evidences from cellular morphology, intracellular structures, and the expression of autophagy-related proteins after the exposure of LNO. We further explored the biological and chemical mechanism about the autophagy. We found that cellular uptake of LNO was time-dependent and then LNO was dissolved in the lysosomes or artificial lysosome fluid due to the acidic environment where metal La and Ni ions were released. Next, the release of metal ions induced ROS production and resulted in oxidative stress that increased lysosomal membrane permeation to induce the autophagy. The possible mechanism about how LaNiO_3_ causes autophagy in the macrophages is shown in **Figure 8**. Moreover, we also evaluated the immune response after the LNO exposure and observed that LNO inhibit the expression of NF-κB, TNF-α, IL-6, and IL-1β at mRNA level, which suggested suppressive effect of LNO on inflammation responses with potential application for the pathogen infection intervene, disease therapy, and tissue repairment. This study revealed the safety and biological effects of perovskite nanomaterials, such as LaNiO_3_ on the macrophages, which will guide the manufacture and design of safe nanomaterials during the production and consumption.

## Data Availability Statement

The raw data supporting the conclusions of this article will be made available by the authors, without undue reservation.

## Author Contributions

Conception and design: LW, XL, and XFG. Development of methodology: LW and XL. Acquisition of data: YW, XJG, DB, YC, and FZ. Analysis and interpretation of data: YW, XJG, FZ, YJ, DB, SH, and YO. Writing, review, and/or revision of the manuscript: LW, XL, and YW. All authors contributed to the article and approved the submitted version.

## Funding

We appreciated the funding from Science and Technology Research Project of Jilin Province Education Department (JJKH20210496KJ), the National Basic Research Program of China (2016YFA0203200, 2020YFA0710702), the National Natural Science Foundation of China (31971322), the Users with Excellence Project of Hefei Science Center CAS (2018HSC-UE004), CAS President’s International Fellowship Initiative (PIFI, 2021PM0059), and the College Students’ Science and Technology Innovation Project in IHEP and UCAS (H95120P0U7). This work was partly supported by the State Key Laboratory of Natural and Biomimetic Drugs, Peking University.

## Conflict of Interest

The authors declare that the research was conducted in the absence of any commercial or financial relationships that could be construed as a potential conflict of interest.
