# Supplementary material for: Induced Autophagy of Macrophages and the Regulation of Inflammatory Effects by Perovskite Nanomaterial LaNiO3
Source: Front Immunol. 2021 Apr 22;12:676773. doi: 10.3389/fimmu.2021.676773 (PMC8100511; doi:10.3389/fimmu.2021.676773)

## Slide 1
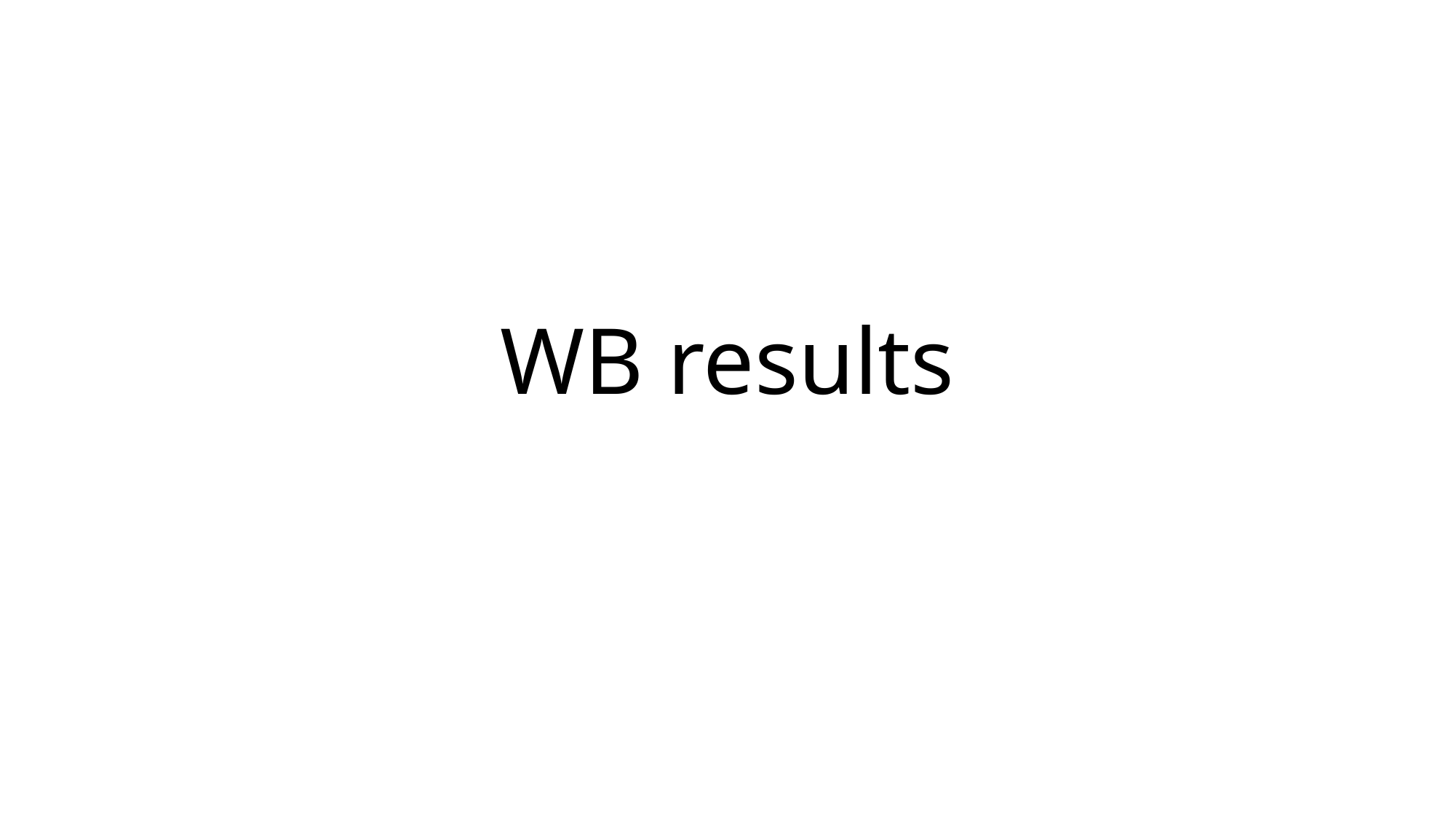

# WB results

## Slide 2
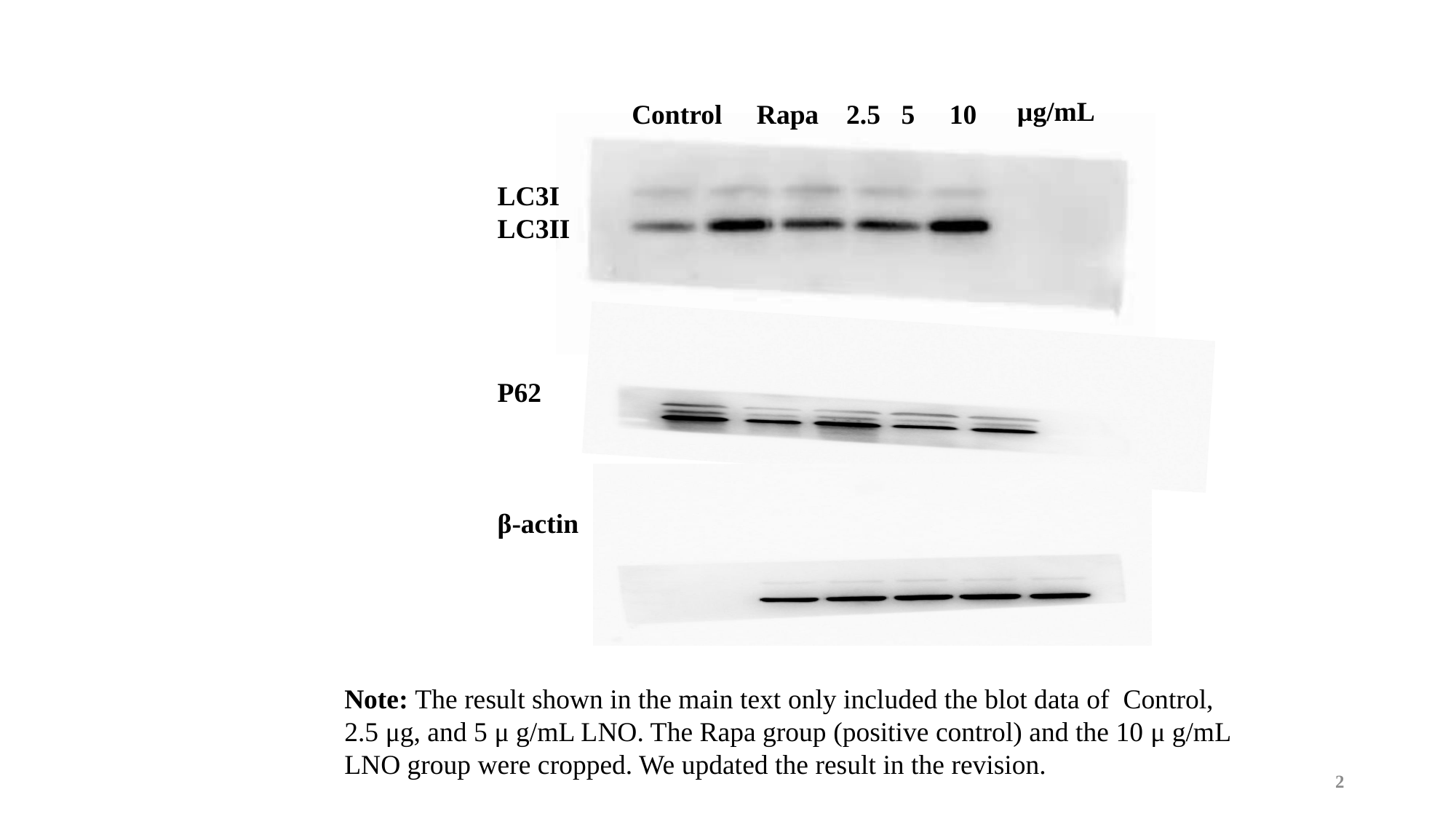

µg/mL
Control Rapa 2.5 5 10
LC3I
LC3II
P62
β-actin
Note: The result shown in the main text only included the blot data of Control, 2.5 μg, and 5 μ g/mL LNO. The Rapa group (positive control) and the 10 μ g/mL LNO group were cropped. We updated the result in the revision.
2

## Slide 3
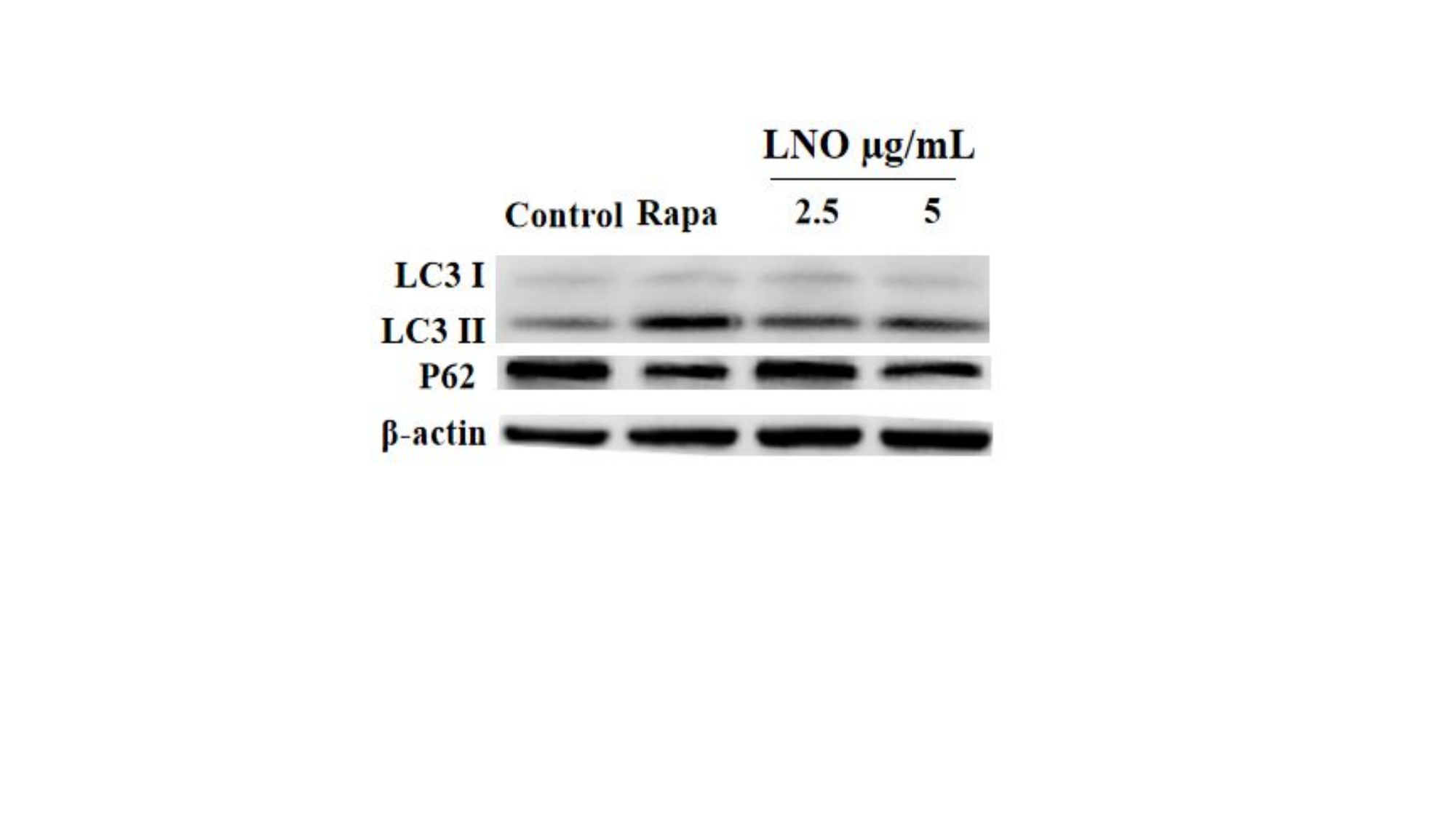

Supplement: Supplementary file 1 [file Presentation_1.pptx]
